# The Influence of the Type of Breathing on the Masticatory Muscle Patterns in Children

**DOI:** 10.3390/medicina60091462

**Published:** 2024-09-06

**Authors:** Daniela Neves-Leal, Antonia M. Caleya, Andrea Martin-Vacas, Nuria E. Gallardo-López, Carlos Gallego

**Affiliations:** 1 Department of Experimental Psychology, Cognitive Processes and Speech Therapy, Faculty of Psychology, Complutense University of Madrid, 28040 Madrid, Spain; dneves@ucm.es (D.N.-L.); carlosgallego@ucm.es (C.G.); 2 Department of Dental Clinical Specialties, Faculty of Dentistry, Complutense University of Madrid, 28040 Madrid, Spain; andrem02@ucm.es (A.M.-V.); negallar@ucm.es (N.E.G.-L.)

**Keywords:** respiration, breathing, masticatory muscle, pediatric dentistry, electromyography

## Abstract

*Background and Objectives:* The aim was to compare the activity of the masseter muscles in children with different types of breathing. *Materials and Methods:* A cross-sectional study was conducted including patients aged 6–12 years with mixed dentition, who came for oral care at the Master’s Degree in Pediatric Dentistry program at the Complutense University of Madrid (UCM), according to inclusion and exclusion criteria. The sample was divided into three groups: nasal breathers without dental alterations (control group), oral breathers with dental malocclusion, and oral breathers with previous orthodontic treatment. An electromyography was performed, and statistic methods were conducted with a 95% confidence to contrast hypothesis. *Results:* A total of 122 children were analyzed and distributed into three groups. The electrical muscle activity of masseters was significantly different between the study groups (*p* < 0.001 for all comparisons). Pairwise comparisons revealed a significantly higher electrical muscle activity in the control group (nasal breathers) during chewing compared to both groups of oral breathers (*p* < 0.001 for both comparisons). Orthodontic treatment decreased electrical muscle activity during isometric contraction in oral breathers compared to nasal breathers (*p* < 0.001), but did not significantly affect electrical muscle activity during chewing. Higher decompensation values were obtained in oral breathers without previous orthodontic treatment compared the other study groups (*p* < 0.001 for both comparisons), although electrical muscle activity values were similar in both groups of oral breathers (*p* > 0.05 for both comparisons). *Conclusions:* Differences in electrical muscle activity between nasal and oral breathers can be confirmed. Oral breathers with and without orthodontic treatment showed lower electrical muscle activity of masseters during chewing than nasal breathers, while at isometric contraction, only oral breathers with previous orthodontic treatment showed lower electrical activity. Higher decompensation values were found in oral breathers without previous orthodontic treatment, in comparison to the control group and oral breathers with previous orthodontic treatment.

## 1. Introduction

Breathing is an involuntary, automatic, and vital process by which oxygen is brought into the blood and carbon dioxide, a metabolic waste product, is expelled from the body. This essential function is classified into nasal, oral, or mixed breathing [1,2]. While nasal breathing (NB) is physiological, oral breathing (OB) is non-functional and should only occur as an adaptive aid to NB. The occurrence of OB can be due to any alteration in the upper airways. Traditionally, the main cause of upper nasal airway obstruction has been attributed to adenoid hypertrophy [3]. However, in recent decades, additional etiological factors have been recognized beyond obstructive causes, including perioral muscle flaccidity, genetic factors, improper oral habits, and the presence of foreign bodies [4,5].

Therefore, when respiratory function is not adequate, alterations in perioral muscle activity can be developed due to alterations in the muscular equilibrium. To measure muscle activity and function, the most used test is electromyography (EMG). Nerves control muscles by sending electrical signals to make them move, and they emit electrical signals while contracting that can be measured in microvolts and in tenths of a second [6,7]. Therefore, EMG indirectly measures the force exerted by muscles by recording action potentials; however, different skin impedance, small variations in the position of electrodes, and fat quantity may influence the results. As the force increases, more action potentials will be recorded [8]. There are two types of EMG: surface EMG (sEMG) and intramuscular EMG. In our field, sEMG is the most used and is the one we will refer to from now on. One of the main characteristics of sEMG is that it is non-invasive, allowing information on muscle function to be obtained under experimental conditions, without danger to the individual [9,10,11,12]. EMG has been developed worldwide, mostly in the field of scientific research. Currently, the methodology used by professionals has been standardized with the aim of adapting the methodology used and reducing the degree of variability of the measurements. For this reason, the SENIAM (Surface Electromyography Non-Invasive Assessment of Muscles) standards have been implemented regarding the protocol for the use of EMG and the placement of the electrodes [13]. In dentistry, sEMG is often used to record activity in functions such as chewing or to record muscle activity in parafunctions such as bruxism. In the case of temporomandibular joint (TMJ) disorders and myofascial pain, it has been used extensively and can be used during orthodontic treatment to address facial pain associated with appliance use [14].

Since the 1980s, several studies on facial muscle activity measurements have consistently shown reduced muscle activity in patients with malocclusion. Pancherz [15] stated that children with class II malocclusion (distocclusion) exhibited lower activity values in the masseters compared to patients with normal occlusion, both during mastication as at maximum intercuspation (MIC). Supporting these findings, Medrano-Montero and Palomino-Truit [16] concluded that the muscle activity in patients with malocclusion is lower than in subjects with normal occlusion, specifically in the masseter muscle. Alvarado [17], using EMG, measured the differences in the masseters’ muscle patterns during mastication and the MIC period, finding no differences in cycle length and chewing interval between children and adults. However, it was verified that adults used the masseters muscles more during chewing than children, and that the chewing rhythm in adults was more stable. A recent study [18] conducted in children and adults measured the activity of the masseter muscles during mastication in patients with normocclusion (class I), mesocclusion (class III), and distocclusion (class II), finding no differences in the electrical potentials of the masseters across the different groups. Studies have also evidenced differences in muscle activity in children with different breathing patterns compared to those with NB [19,20,21]. In the field of Otorhinolaryngology, Ferla et al. [22], in agreement with previous authors [15,16], concluded that OB affected electrical activity during chewing and MIC, and presented asymmetry. 

Referring to the studies that investigated the muscular changes produced using orthodontic treatments, Tartaglia et al. [23] evaluated children who were undergoing functional orthodontic treatment through sEMG. The results concluded that, after six months of treatment, the functional devices provided significant structural differences. However, there were no differences at the muscular level. In agreement with the authors, Castañeda et al. [24] analyzed changes in electrical activity during the different phases of orthodontic treatment, verifying that muscular changes decreased randomly and inconsistently during the phases of orthodontic treatment. Contrary to previous studies, Viveros [25] concluded that the changes produced using the orthopedic devices had also produced neuromuscular changes towards balance. 

The main objective of the present study was to compare the tone and activity of the masseter muscles in children with OB who had not received orthodontic treatment, children with OB who had received it, and children with NB in normocclusion. The research hypothesis is based on affirming that the muscle activity of masseters in isometric sustained contraction (strongly occluding with the posterior teeth) and during mastication are greater in children with physiological nasal breathing compared to oral breathers. 

## 2. Materials and Methods

This cross-sectional analytical and observational study was approved by the Institutional Ethics Committee of the Deontological Commission of the Doctoral Program in Psychology of the Complutense University of Madrid (UCM) in Spain, in accordance with the criteria for research involving human subjects (Code 2018/19-003). The research was conducted ethically, with all study procedures performed in accordance with the requirements of the World Medical Association’s Declaration of Helsinki.

### 2.1. Population and Sample Selection

Due to the heterogeneity in published studies, it was not possible to estimate the sample size according to previous authors. The sample size was calculated theoretically with G*Power 3.1 software (version 3.1.9.7., Düsseldorf, Germany) using the ANOVA procedure (fixed effect), with special, main effects and interactions [26,27]. For three groups, with an alpha error of 0.05 and an effect size of 0.40 (large), 84 patients (28 for each group) were required to have a statistical power of 95% in a quantitative variable. To accommodate potential issues such as collaboration problems with children or interferences during measurements, the sample size was increased by a third, resulting in a final sample size of approximately 120 patients, with approximately 40 patients per group.

The sample was selected among child patients who met the following inclusion criteria: (1) children aged 6–12 years, (2) a clinical diagnosis of oral breathing (with or without previous orthodontic treatment), (3) mixed dentition (complete eruption of first permanent molars and intercuspation with the antagonist), and (4) came for oral care at the Master’s Degree in Pediatric Dentistry at UCM between 2020 and 2022. The exclusion criteria included the following: (1) non-Caucasian facial typology, (2) children with variations in facial pattern (both at the bone growth level and in their facial muscle characteristics), (3) patients with a history of facial or dental trauma, (4) children with orofacial deformities or congenital or hereditary disorders, (5) children with systemic neurological or muscular disease, (6) syndromic patients, (7) children with disturbances in language development, (8) children with chronic or regular medication, (9) children with severe crown destruction or premature tooth loss, (10) children with transverse or vertical malocclusion, (11) patients with orofacial and/or TMJ pain, (12) children who had previously received speech myofunctional therapy, and (13) patients who were wearing orthodontic appliances during the study. 

Children were diagnosed by pediatric dentists during routine dental examinations, which include the assessment of breathing type and TMJ status. Children with TMJ pain (referred to by the patient at rest or movement) or joint sounds were diagnosed of TMJ disease and excluded for the evaluation. The reason for excluding these patients was that orofacial pain can interfere with masticatory condition and symmetry, and referred muscular pain could potentially alter the sEMG results. The assessment of breathing by the pediatric dentist is the first step of the protocol followed at our institution, serving as a screening tool, followed by referring children with breathing difficulties to the otolaryngology and speech therapy services. This assessment includes the evaluation of breathing following the protocol proposed by de Félicio et al. [28,29,30], through the observation of the child. Facial pattern assessment involves comparing the proportion of the facial thirds. When these proportions are harmonious, it is a mesofacial pattern, being called brachyfacial or dolichofacial when the lower facial third is, respectively, decreased or increased in size. Children categorized in the “orthodontically treated” group have received dental appliances, excluding corrective orthodontics such as multi-brackets or clear aligners.

After applying the inclusion and exclusion criteria, two groups of patients were formed using non-probabilistic consecutive sampling based on whether they had undergone previous orthodontic treatment. A control group of nasal breathers without structural alterations or upper airways patency without previous orthodontic treatment, matched for age and sex, was also included. 

Dental malocclusion was determined using Angle’s classification, stating that the first permanent molar and canine are the most stable teeth of the permanent dentition, and act as a reference for occlusion diagnosis. Angle’s classification categorizes malocclusion based on the mesiodistal relationship of permanent teeth, dental arches, and jaws, depending mainly on the mesiodistal positions of the upper and lower first permanent molars. According to this classification, a normocclusive relationship between the molars is determined when the mesiobuccal cusp of the maxillary first permanent molar articulates with the buccal groove of the mandibular first permanent molar. Angle class I malocclusion denotes misaligned teeth (i.e., rotations) but a correct mesiodistal relationship between the upper and lower molars; Angle class II and III malocclusion indicate, respectively, a distal or mesial positioning of the lower molar relative to its upper equivalent. The Angle’s classification was applied only to assess the relationship of the first permanent molar [31] as the sample was in mixed dentition.

The final sample was constituted as follows:

- NG Group (control sample): Children with NB with dental normocclusion and mixed dentition who had not undergone any orthodontic treatment.
- OB− Group: Children with oral or mixed breathing and with dental malocclusion who had not received any orthodontic treatment.
- OB+ Group: Children with oral or mixed breathing and with dental malocclusion who had received orthodontic treatment, subsequently presenting normal dental occlusion.

The participants and their legal guardians were fully informed about the study and signed the informed consent. Once the study groups were assigned, the relevant information was blinded to the operator to prevent study biases during the measurements. 

### 2.2. Study Procedure

Record-taking, which took approximately 30 min, was conducted in a single session for each patient, performed by a myofunctional speech therapist (D.N.-L) and a pediatric dentist (N.E.G.-L). First, children underwent an examination following the validated OMES protocol (Orofacial Myofunctional Evaluation with scores) [28,29,30], implemented to verify the type of breathing of the included patients. Four parameters were evaluated: (1) orofacial muscle posture at rest, (2) mobility of orofacial structures, (3) respiratory function, (4) swallowing and chewing. Each participant underwent five intraoral photographs—frontal occlusion, right side (R) and left side (L), upper and lower arch—and four extraoral photographs—frontal, at rest and smiling, and right lateral, at rest and smiling.

Muscle assessment was conducted with sEMG using a NeuroTrac^®^ MyoPlus 2/4 PRO Electromyograph (Neurotrac, Southampton, UK). The technical specifications of the electromyograph, according to manufacturer information (https://www.tiendaneurotrac.com/manuales/myoplus2pro.pdf, accessed on 10 November 2022), include dual-channel EMG functionality, with an EMG range of 0.2–2000 μV RMS (extended), a sensitivity of 0.1 μV RMS, and an accuracy of 4% of μV ± 0.3 μV indications at 200 Hz. Regarding the selective band filter of 3 dB, it can be either wide (18 Hz ± 4 Hz to 370 Hz ± 10%—reading below 235 microvolts 10 Hz ± 3 Hz to 370 Hz ± 10%—reading above 235 microvolts) or narrow (100 Hz ± 5% to 370 Hz ± 10% 1.5 Notch filter: 50 Hz–33 dbs 0.1% accuracy). 

Gel surface electrodes (Kendall™ Arbo™ ECG Electrodes H124SG, 30 mm × 24 mm (Coviden Spain Sl, Cornella de LLobregat, Barcelona, Spain) with a single adhesive side and non-irritating and hypoallergenic hydrogel, latex-free and suitable for children’s skin type, were used. The electrical characteristics of electrodes according to ANSI/AAMI EC 12, the average measured before packing, included an ACZ I impedance of 220 Ohm, a DC offset voltage of 0.2 mV, an SDR of 11 mV, a Slope of 0.2 mV/s, a COIIN of 4µV and a bias current tolerance of 6 mV (Kendall™ ECG Electrodes Product Data Sheet Arbo™ H124SG Ref. Code: 31.1245.21). The hydrogel can reduce noise and provides better resolution in measurements. The study adhered to the SENIAM protocol standards [13] and was consistently performed by the same operator (D.N.-L.) who was trained in EMG technique. First, the entire procedure was thoroughly explained to the parents/guardians as well as to the participating children. The recordings were always taken in the same room, isolated, made without noise and always with the same device, and all electronic devices that could cause interference were eliminated. 

**Figure 1:**
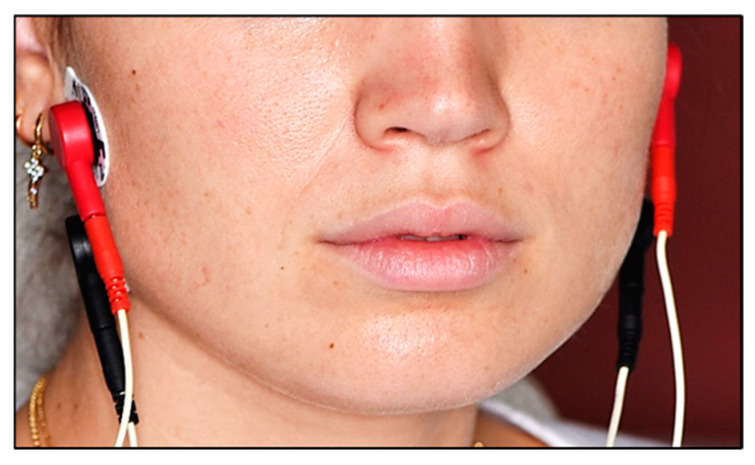
Illustration of electrode position on each child’s face.

The children were seated with their backs straight, with the Frankfurt plane parallel to the floor, using an ergonomic dental chair and anatomically correct positioning, allowing for proper mastication without the risk of dysphagia and/or choking. Before placing the electrodes, the entire area was cleaned with a cotton disk soaked in ethyl alcohol. Four channels of the electromyography device were used. So, four surface electrodes were placed on each child’s face (Figure 1), two on the patient’s right side and the other two on the left side. For each side, one was located at the inferior portion of the masseter (angle of the mandible) and the other, 20 mm from the first, located at a point on the line that connects the mandibular angle with the wing of the nose (upper portion of the masseter). The reference electrode was placed on the patient’s wrist and was selected due to its minimal muscle mass to avoid reading biases. Once the electrodes had been placed, the EMG program was selected on the device and a test session was carried out to train the patient.

**Figure 2:**
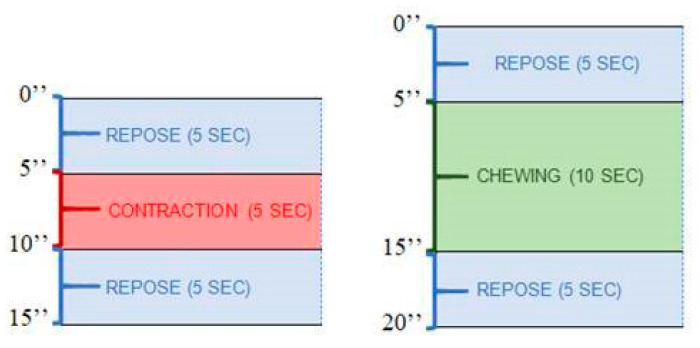
Electromyographic measurement protocol for isometric contraction and chewing activity.

The measurement protocol was composed of two phases, a first one with an isometric contraction and a second one with chewing activity (Figure 2). Firstly, the muscle activity in the sustained or isometric contraction of the masseters (ICM) was recorded, and the data were registered separately for the R and L sides. The total test lasted 15 s for each side. The secondary, chewing activity of the masseters was recorded for both sides simultaneously, using the R and L sides of the electromyograph at the same time (CAM). To simulate chewing, masticatory rollers placed in the molar area were used and the child had to remain with their lips closed. This second test lasted 20 s and had to be performed applying a comfortable, non-forced chewing for the patient. 

An asymmetry index was additionally calculated from the differences in the output of the side channels (R and L) for each of the measurements, obtaining the decompensation values for ICM (D-ICM) and CAM (D-CAM). The values of the dependent variables were derived from EMG recordings in ICM and D-ICM between the R and L, as well as CAM and D-CAM between the masticatory sides. For the ICM variable, the mean ICM values were calculated in microvolts in the contraction period of 5 s, between rest or repose periods of 5 s. The D-ICM corresponding to the asymmetry between both sides was also calculated and defined as the difference between channels R and L. The higher the absolute value, the higher the degree of imbalance or asymmetry. For the CAM variable, the average of the masticatory peaks during a 10 s chewing was used as the measure. A peak was defined as any record that exceeded both the preceding and following values and was greater than the mean of the period. The average of the masticatory peaks was taken in microvolts between the 5–15th seconds between the initial and final rest periods. 

### 2.3. Data Analysis

Descriptive statistics were performed, with the calculation of the mean and median of CAM-R, CAM-L, CAM, and D-CAM. To analyze the adjustment to a normal distribution, asymmetry and kurtosis indexes were conducted, finding that the variables did not meet normality criteria in all cases. Due to this, the data were analyzed using non-parametric statistics. The analyses were carried out on the values in microvolts in ICM, D-ICM, CAM, and D-CAM. To analyze the differences between the groups, the Kruskal–Wallis test was used. Once the existence of differences between the groups was verified, the Mann–Whitney U test was used for pairwise comparisons, and Bonferroni correction was applied to adjust *p* values for post hoc comparisons. The Cliff’s Delta statistic (δ) was chosen as the estimator of the effect size as it is more appropriate when the criteria of homogeneity of variance or normality are not met. The effect size of the Cliff’s Delta statistic was interpreted based on Cohen’s norms [32]; an effect size of 0.2 was considered as small, 0.5 as a medium size, and a greater than or equal to 0.8 as a large effect size. Data analysis was conducted with the SPSS 22^®^ software (version 22.0, Armonk, NY, USA) for Windows, with a confidence level of 95% (*p* ≤ 0.05), and asymptotic or bilateral significance.

## 3. Results

**Figure 3:**
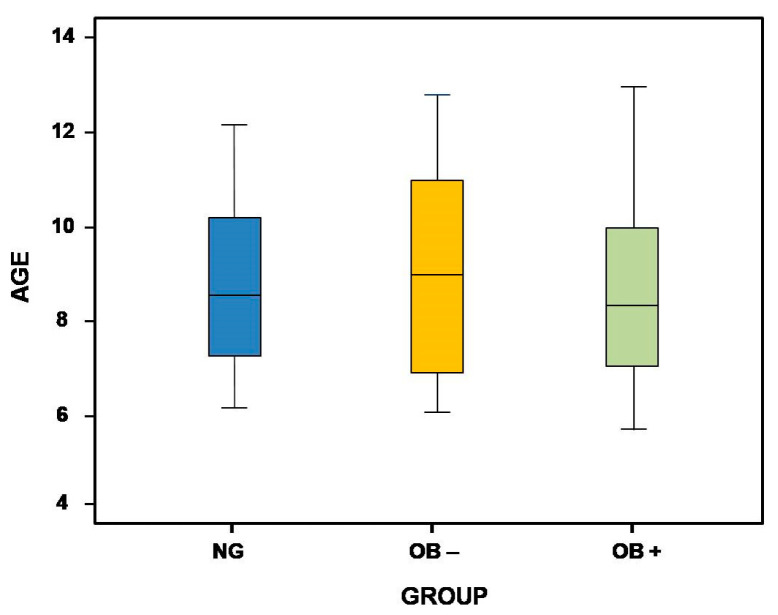
Boxplot graph of age in the three study groups. NG, nasal breathers group; OB−, oral/mixed breathing, with dental malocclusion who had not received any orthodontic treatment; OB+, oral/mixed breathing with dental malocclusion, who had received orthodontic treatment.

The final sample consisted of 122 participants, distributed in three groups: 40 children in the control group (mean age = 8.6 ± 1.74 years), 40 children in OB+ Group (mean age = 8.81 ± 2.22 years), and 42 children in OB− Group (mean age = 8.62 ± 2 years). The female/male ratio was 23/17 in the control group, 22/20 in OB− group, and 23/17 in OB+ group. The age and sex homogeneity between the three study groups was verified, with no significant differences for age (*χ²* = 0.102, *p* = 0.95) (Figure 3) nor sex (*χ²* =0.290, *p* = 0.865). 

Regarding the study variables (ICM, D-ICM, CAM, and D-CAM), descriptive statistics, including the mean and standard deviation (SD), were calculated (Table 1). Significant differences were found between the three study groups (*p* < 0.001 for all comparisons). In relation to the differences between the normal and oral breathers groups, both the ICM and CAM values were higher for NG and OB− than OB+. 

Pairwise comparisons were conducted (Table 2), and a significantly higher electrical muscular activity was found in the NG compared to OB− group in CAM, D-ICM, and D-CAM (*p* < 0.001 for all comparisons); however, the differences were not statistically significant in ICM (*p* = 0.117). These findings point to differences between the right and left masticatory sides, both in ICM and during the period of chewing, as well as differences in global CAM. Furthermore, electrical muscular activity showed significantly higher values in the NG compared to the OB+ group in ICM and CAM (*p* < 0.001 for both comparisons), with no significant differences in D-ICM (*p* = 0.885) or D-CAM (*p*= 0.736). The comparison between oral breathers groups was carried out, finding significantly higher electrical muscular activity values in the OB− group compared to OB+ group in ICM (*p* = 0.001), in D-ICM, and in D-CAM (*p* < 0.001 for both comparisons); however, the differences were not significant in CAM (*p* = 0.099). 

## 4. Discussion

This study was conducted on 122 children with different types of breathing; comparing previous interceptive orthodontic treatments is of significant scientific interest for both speech therapists and dentists, as they provide an objective basis for contrasting neuromuscular patterns as indicators of potential functional alterations. Furthermore, the sample size and age of the patients are notably advantageous compared to all previously existing studies on this topic or similar ones in the field of sEMG in child patients of developmental age, increasing the reliability of the study. The large sample size of 122 patients evaluated in this study is at least three times larger than the samples used in similar studies, which range from 29 to 45 subjects [16,18,22].

Our results confirm that indeed, the muscle activity of masseters in contraction was significantly higher in NG in comparison to the OB+ group, but no significant differences were found related to the OB− group. However, although the findings in relation to the OB- group were not significant, the mean values in NG were higher. In this line, Ferla et al. [22] compared masticatory activity in oral and nasal breathers, verifying that the muscular electrical activity during mastication and MIC was lower in the OB group compared to the control group. However, according to the proposed hypothesis, the differences found in the MIC between the NG and the OB+ indicated a significative greater muscular electrical activity in the MIC in the NG. The average muscular electrical activity in MIC in the OB+ group is very low, even leading to significant differences in comparison to the OB− group. This may be attributed to the reduction in muscle activity caused by orthodontic treatment and occlusal changes. Rodríguez Castañeda et al. [24] verified using sEMG that the muscular electrical in MIC activity during the different phases of an orthodontic treatment decreased randomly during the treatment phases. On the other hand, Viveros Martínez [25] evaluated, using EMG, the activity of the masticatory muscles in children with class II malocclusion treated with orthodontics, stating that there are significant changes in masseter muscles after orthodontic treatment, both at rest and in MIC. This might be due to a neuromuscular adaptation, aiming at restoring balance, secondary to the dental and occlusal changes. According to our results, Viveros Martínez [25] stated that isometric contraction would be symmetrical in the NC and the OB+ group, while it would be asymmetrical in the OB− subjects. The authors affirm that this difference could be a result of the increased functional dominance of one facial side, associated with a preferred and/or exclusive unilateral chewing pattern, that increases muscle tone on the working and reduces side tone in the contralateral muscles, the swinging side.

Furthermore, our results indicated that there are no significant differences in the absolute degree of decompensation (asymmetry) between the NG and both OB+ groups. Conversely, and as expected, the differences in decompensation between the NG and the OB− group, and between both OB groups, were significant. Nasal breathers present a lower degree of decompensation, as well as the OB+ group, and therefore there is symmetry between both masticatory sides, most probably due to a balancing in the occlusal contacts. On the other hand, the OB− group presented a high decompensation between both sides, and greater variability and dispersion in the masseters electrical activity exerted between both sides is observed. 

Regarding CAM, significantly higher mean values have been found between the NG and both groups OB groups, with no significant differences between the OB+ and OB− groups. Therefore, it can be elucidated that children with oral breathing, who had orthodontic treatment, have lower chewing muscle electrical activity, compared to children who present normative development and breathing. The greater CAM observed in the NG group compared to both OB groups may be primarily attributed to oral breathing in the latter. Oral breathing leads to general orofacial muscle weakening, particularly in the masticatory muscles, due to the prolonged maintenance of an inadequate open-mouth posture, a lowered tongue, and the absence of a lip seal. In OB-, in addition, the decrease in CAM can be increased by the greater instability of the chewing pattern associated with unilateral chewing and/or unstable mandibular movements. According to our results, Pancherz [15] affirmed that children with class II malocclusion had lower activity values both during chewing and at MIC. On the other hand, Medrano-Montero and Palomino-Truit [16] concluded that the masseter muscle had significantly lower values in subjects with malocclusion compared to subjects with normal occlusion. Ferla et al. [22] verified that oral breathing decreased muscle electrical activity during mastication and MIC. According to the obtained results, in the OB+ group, the decrease in CAM due to oral breathing could be increased by the change in chewing habits, secondary to occlusal correction obtained with orthodontics. Orthodontic appliances can lead to the selection of softer foods that facilitate the chewing process, ultimately contributing to a long-term decrease in muscle activity. This preference may be related to the difficulty in forming the food bolus with harder foods and the discomfort that orthodontic appliances may cause.

Related to symmetry in contralateral masticatory sides, according to the obtained results, Ferla et al. [22] stated that masticatory symmetry in nasal or oral breathers without malocclusion is due to bilateral chewing, and, therefore, asymmetry or decompensation in oral breathers with malocclusion is secondary to unilateral chewing. Furthermore, functionally, both nasal and oral breathers with an orthodontically corrected malocclusion would exhibit a more stable, balanced, and compensated occlusion compared to children with oral breathing and non-corrected malocclusion, due to a greater number of dental contacts and functional symmetry, which both contribute to a greater competence during chewing. Orthodontic appliances can modify the orofacial characteristics of oral breathing, increasing structural symmetry, but without modifying electrical muscle activity, because the function is not well established. According to Begnoni et al. [33], myofunctional therapy conducts favorable muscle changes after the correction of atypical swallowing, complementing neuromuscular changes obtained with orthopedic devices, generating a tendency towards muscle symmetry of masseters and balancing occlusion. Therefore, if there is a deficit in muscle activity or bilateral muscle balance or both, myofunctional therapy would be an appropriate treatment option. 

Given the increasing prevalence of Obstructive Sleep Apnea (OSA) in pediatric patients, it would be valuable to investigate the underlying causes of OB. Our preliminary results, which show differences depending on the type of breathing, suggest that it would be of significant scientific interest to explore whether specific muscular alterations are present in children with OSA. Thus, pre-post longitudinal studies would be interesting to see the evolution of masticatory muscle activity in this group of children. The assessment and treatment of children with OSA should always be monitored by a multidisciplinary team (otorhinolaryngologists, speech therapists and dentists, among others) in accordance with the most current scientific evidence [34]. 

The main limitation of this study is probably the cross-sectional design; therefore, causal inferences cannot be made, although the exclusive theoretical calculation of the sample size due to the heterogeneity in the characteristics of previous studies may affect the generalization of the obtained results and the achievement of statistical significance for some comparisons. To improve extrapolation to the general population, broader studies should be conducted, including transverse or vertical malocclusions, to improve the knowledge of the muscle patterns alteration related to malocclusions. Obtaining EMG measurements before and after performing orthopedic treatment in oral breathers would provide more reliable results regarding the modification of the muscle pattern in these patients. Some biases like little differences in skin impedance or electrodes position cannot be controlled. It is important to consider that during the ages of 6–12 years, physiological dental replacement occurs, which may lead to muscular compensations during the eruptive process. However, the authors believe that this does not affect the results, as it would impact both study groups equally, given the homogeneity in age among the groups of children studied. There is a lot of variation in our results that suggests that the method of measuring CAM is not specific and/or sensitive in pediatric patients, which makes it necessary to carry out more studies that analyze the relationship between breathing and facial muscle pattern. 

Despite the limitations encountered, we believe that the obtained results can serve as a valuable starting point for the analysis and treatment of muscular disorders in children with dental malocclusion. Additionally, it can stimulate collaborative efforts between dentists and speech therapists in the comprehensive treatment of patients. Muscular stability, achieved through myofunctional treatment, will help maintain the occlusal results obtained after orthodontic–orthopedic treatment and prevent recurrences.

## 5. Conclusions

Within the limitations of this study, differences in the electrical muscle activity of masseters between nasal and oral breathers, both isometrically and during chewing activity, can be confirmed. Electrical muscle activity during isometric contraction was significantly higher in the control group compared to oral breathers with previous orthodontic treatment, with no differences compared to oral breathers without orthodontic treatment. The electrical muscle activity of masseters during chewing was higher in the control group in comparison to both groups of oral breathers, independently of previous orthodontic treatment. Electrical muscle activity values of masseters showed no significant differences in oral breathers with and without previous orthodontic treatment. Furthermore, higher decompensation values were found in oral breathers without previous orthodontic treatment in comparison to normal group and oral breathers with previous orthodontic treatment.

## Figures and Tables

**Table 1:** Descriptive statistics in the three study groups and significance of the differences between groups in the four variables tested.

	Group	Mean	SD	Median	IQR	*χ²*	*p* Value *
ISOMETRIC CONTRACTION OF MASSETERS (ICM)	NG	103.24	55.08	84.05	51.46	24.06	<0.001
	OB−	83.56	37.59	75.33	58.69		
	OB+	61.07	39.73	58.37	32.78		
DECOMPENSATION IN ISOMETRIC CONTRACTION OF MASSETERS (D-ICM)	NG	13.14	17.16	7.54	13.39	37.29	<0.001
	OB−	33.71	25.67	31.33	20.12		
	OB+	12.39	17.46	7.09	14.62		
CHEWING ACTIVITY OF MASSETERS (CAM)	NG	159.66	54.75	146.34	40.30	37.82	<0.001
	OB−	108.10	58.05	100.29	65.50		
	OB+	89.77	49.90	74.74	65.60		
DECOMPENSATION IN CHEWING ACTIVITY OF MASSETERS (D-CAM)	NG	14.66	17.92	8.69	14.18	30.36	<0.001
	OB−	40.40	34.41	33.59	34.04		
	OB+	14.19	15.60	10.63	15.21		

* *p* value for Kruskal–Wallis test. Significance *p* < 0.05. SD. Standard deviation. IQR. Interquartile range. NG. Nasal breathing group. OB−. Oral/mixed breathing group with dental malocclusion, who had not received any orthodontic treatment. OB+. Oral/mixed breathing group with dental malocclusion, who had received orthodontic treatment.

**Table 2:** Pairwise comparison between study groups.

	NG-OB−		NG-OB+		OB−-OB+	
	*p* Value *	δ	*p* Value	δ	*p* Value	δ
ISOMETRIC CONTRACTION OF MASSETERS (ICM)	0.117	0.20	<0.001	0.63	0.001	0.41
DECOMPENSATION IN ISOMETRIC CONTRACTION OF MASSETERS (D-ICM)	<0.001	0.65	0.885	0.02	<0.001	0.70
CHEWING ACTIVITY OF MASSETERS (CAM)	<0.001	0.61	<0.001	0.73	0.099	0.21
DECOMPENSATION IN CHEWING ACTIVITY OF MASSETERS (D-CAM)	<0.001	0.60	0.736	0.04	<0.001	0.61

* *p* value for Mann–Whitney U test. Significance *p* < 0.05. δ. Cliff’s Delta statistic. NG. Nasal breathing group. OB−. Oral/mixed breathing group with dental malocclusion, who had not received any orthodontic treatment. OB+. Oral/mixed breathing group with dental malocclusion, who had received orthodontic treatment.

## Data Availability

The datasets presented in this article are not readily available because are part of a doctoral thesis that has not yet been defended. Requests to access the datasets should be directed to dneves@ucm.es.

## Abbreviations

CAM	Chewing Activity of Masseters
D-ICM	Decompensation Isometric Contraction of Masseters
D-CAM	Decompensation Chewing Activity of Masseters
EMG	Electromyography
ICM	Isometric Contraction of Masseters
L	Left side
MIC	Maximum Intercuspation
NB	Nasal Breathing
NG	Nasal Group
OB	Oral Breathing
OB−	Children with oral or mixed breathing, with dental malocclusion, who had not received any orthodontic treatment
OB+	Oral or mixed breathing, with dental malocclusion, who had received orthodontic treatment
OMES	Orofacial Myofunctional Evaluation with Scores
OSA	Obstructive Sleep Apnea
R	Right side
δ	Cliff’s Delta statistic
sEMG	Surface EMG
SENIAM	Surface ElectroMyoGraphy for the Non-Invasive Assessment of Muscles
UCM	Complutense University of Madrid
